# Functional Polymers as Innovative Tools in the Delivery of Antimicrobial Agents

**DOI:** 10.3390/pharmaceutics14030487

**Published:** 2022-02-23

**Authors:** Umile Gianfranco Spizzirri

**Affiliations:** Department of Pharmacy, Health and Nutritional Sciences, University of Calabria, 87036 Rende, Italy; g.spizzirri@unical.it; Tel.: +39-0984-493298

Ubiquitous microorganisms such as bacteria, viruses, algae, and fungi induce several infectious diseases, representing crucial health challenges worldwide, due to increased antimicrobial resistance, high antimicrobial cost and adverse effects. The association between the growth of resistance mechanisms and the progress of new antimicrobial molecules is of great concern to public health. Therefore, it is important to consider innovative ways to face this resistance problem, such as the application of nanotechnologies, which are interesting alternatives to traditional antibiotic development approaches [1]. In the last decade, to overcome these issues and to successfully fight infections, new macromolecular carriers able to significantly improve the efficiency of antimicrobial species were proposed [2]. In this regard, antimicrobial delivery systems, properly entrapping these bioactive molecules, avoid their degradation by also decreasing the frequency of their administration [3]. This Special Issue explores different topics concerning recent progresses in the synthesis and characterization of suitable innovative macromolecular systems, proposed as carriers of specific antimicrobial molecules, to be employed in the biomedical and pharmaceutical fields.

Nowadays, the treatment of infections produced by multidrug-resistant bacteria expects as a last resort the employment of polymyxins, a potent class of peptide antibiotics. In order to overcome the limitations related to the nephro- and neurotoxicity of these compounds and to improve their antimicrobial activity and target specificity, several novel polymyxin derivatives are being developed, involving structural modifications of the peptides [4]. In this regard, conjugation to water-soluble polymers provides many advantages, such as sustained plasma half-life and reduced toxicity [5]. Nanoliposomes coated with highly deacetylated chitosan and colistin were prepared and characterized using dynamic light scattering and zeta potential measurements [6]. The antimicrobial activity of these formulations increased by four-fold against *P. aeruginosa*, but did not have any antimicrobial activity against multidrug-resistant bacteria. Alternatively, a low-molecular-weight alginate oligosaccharide, able to inhibit bacterial growth, adherence and biofilm development, and which potentiates the activity of antibiotics against Gram-negative multidrug-resistant pathogens, was proposed to create a bi-functional antibiotic polymer [7]. Hydroxyl and carboxyl functional groups in this oligosaccharide were successfully employed to prepare a colistin-based adduct that, combining the antimicrobial properties of both the antibiotics, was able to inhibit multidrug-resistant species, as well as Gram-negative *P. aeruginosa*, both in in vitro and in vivo experiments.

Alternatively, advanced micellar carriers were proposed for the encapsulation of norfloxacin, one of the most-used antibiotics from the class of fluoroquinolones, showing antibacterial activity against both Gram-positive and Gram-negative bacteria, and used to treat a large variety of urinary or respiratory tract infections [8]. Specifically, novel polymeric micelles based on Pluronic F127 and Cremophor EL were investigated as drug carriers for norfloxacin, exhibiting good activity against *S. aureus*, *E. faecalis*, and *E. coli*, while *P. aeruginosa* displayed low sensitivity to norfloxacin in all tested systems [9].

The involvement of nanoparticles in the preparation of new effective carriers to deliver antimicrobial agents was also deeply investigated [10]. This Special Issue explored the possibility of employing poly (methyl methacrylate) nanoparticles as carriers of microbicides, such as quaternary ammonium surfactants. In particular, antibacterial performances of the synthesized carriers, based on cetyl trimethyl ammonium bromide or dioctadecyl dimethyl ammonium bromide and poly (methyl methacrylate), were analyzed [11]. Antimicrobial properties were assessed by viability curves of *E. coli*, *S. aureus* and *C. albicans*. Significant inhibition against bacteria and yeast was observed only for cetyl trimethyl ammonium bromide, while dioctadecyl dimethyl ammonium bromide just displayed fungicidal activity against *C. albicans*.

Encapsulating antimicrobial species into nanoparticles and incorporating these nanoparticles with bio-based film to fabricate nanocomposite films represents an innovative and effective strategy to control the release of the active molecules. Specifically, a long-term antibacterial film nanocomposite composed by zein film and cinnamon essential oil-loaded MCM-41 silica nanoparticles was evaluated against *S. aureus* [12]. The results clearly highlighted that the addition of silica nanoparticles significantly improve the mechanical properties of zein films, prolonging, at the same time, the antibacterial effect of the cinnamon essential oil.

Alternatively, hydrogels appear to be excellent candidates as antibiotic delivery platforms slowing down the progression of bacterial resistance to antibiotics [13]. In this context, Carreno et al. (2020) proposed hydrophilic macromolecular systems synthesized by cross-linking polyvinyl alcohol and aliphatic dicarboxylic acids, such as glutaric acid, adipic acid or succinic acid to specific release linezolid, a chemotherapeutic agent able to treat bacterial resistance accepted for the treatment of complex skin infections [14]. The antibacterial tests against *E. faecium* bacterial strain confirmed that the sustained release of linezolid from a glutaric acid-based hydrogel showed the best antibacterial activity.

This Special Issue was completed by three reviews providing an overall view on the use of nanotechnology in the transport and release of antimicrobial molecules. In particular, Wang et al. (2021) analyzed the recent advances in the highly efficient delivery of antimicrobial agents by polymeric nanomaterials such as dendrimers, micelles, nanofibers, nanogels and vesicles [15]. The authors concluded that the versatility of the polymeric nanomaterials provided several benefits, showing significant potential in a wide variety of biomedical applications, such as fighting multidrug-resistant bacteria, wound healing and anti-biofilm. On the contrary, the other two reviews focused their attention on the delivery of specific antimicrobial agents. Spizzirri et al. (2021) discussed the most innovative strategies to synthesize nanodevices able to release antimicrobial natural extracts originating from herbs, plants, and agro-food waste by-products [16]. Finally, Jancic and Gorgieva highlighted the structure and properties of plant origin bromelain and antimicrobial peptide nisin, analyzing their mechanisms of action and the immobilization strategies involving macromolecular systems, in order to expand their application in the pharmaceutical and biomedical fields [17].

In conclusion, the challenges for the large-scale fabrication and translation from the bench to clinical trials of these polymeric systems to fight the multidrug resistance of several dangerous pathogens will represent a major focus in the coming decades. However, to overcome this issue, novelty from chemists and engineers, as well as regulatory policies able to simplify access to trials and patients are required. 
